# FUS-DDIT3 Fusion Oncoprotein Expression Affects JAK-STAT Signaling in Myxoid Liposarcoma

**DOI:** 10.3389/fonc.2022.816894

**Published:** 2022-02-03

**Authors:** Soheila Dolatabadi, Emma Jonasson, Lisa Andersson, Manuel Luna Santamaría, Malin Lindén, Tobias Österlund, Pierre Åman, Anders Ståhlberg

**Affiliations:** ^1^ Sahlgrenska Center for Cancer Research, Department of Laboratory Medicine, Institute of Biomedicine, Sahlgrenska Academy at University of Gothenburg, Gothenburg, Sweden; ^2^ Wallenberg Centre for Molecular and Translational Medicine, University of Gothenburg, Gothenburg, Sweden; ^3^ Region Västra Götaland, Department of Clinical Genetics and Genomics, Sahlgrenska University Hospital, Gothenburg, Sweden

**Keywords:** cancer stem cells, CD44, FET sarcomas, FUS-DDIT3, JAK-STAT signaling, myxoid liposarcoma

## Abstract

Myxoid liposarcoma is one of the most common sarcoma entities characterized by FET fusion oncogenes. Despite a generally favorable prognosis of myxoid liposarcoma, chemotherapy resistance remains a clinical problem. This cancer stem cell property is associated with JAK-STAT signaling, but the link to the myxoid-liposarcoma-specific FET fusion oncogene *FUS-DDIT3* is not known. Here, we show that ectopic expression of FUS-DDIT3 resulted in elevated levels of STAT3 and phosphorylated STAT3. RNA sequencing identified 126 genes that were regulated by both FUS-DDIT3 expression and JAK1/2 inhibition using ruxolitinib. Sixty-six of these genes were connected in a protein interaction network. Fifty-three and 29 of these genes were confirmed as FUS-DDIT3 and STAT3 targets, respectively, using public chromatin immunoprecipitation sequencing data sets. Enriched gene sets among the 126 regulated genes included processes related to cytokine signaling, adipocytokine signaling, and chromatin remodeling. We validated CD44 as a target gene of JAK1/2 inhibition and as a potential cancer stem cell marker in myxoid liposarcoma. Finally, we showed that FUS-DDIT3 interacted with phosphorylated STAT3 in association with subunits of the SWI/SNF chromatin remodeling complex and PRC2 repressive complex. Our data show that the function of FUS-DDIT3 is closely connected to JAK-STAT signaling. Detailed deciphering of molecular mechanisms behind tumor progression opens up new avenues for targeted therapies in sarcomas and leukemia characterized by FET fusion oncogenes.

## Introduction

More than 25 types of sarcoma and leukemia are characterized by fusion oncogenes formed between one of the FET genes (*FUS*, *EWSR1*, or *TAF15*) as 5′ partner and one of more than 30 alternative transcription factor-coding genes as 3′ partner (1, 2). The FET fusion oncoproteins act as abnormal transcription factors *via* interactions with the SWI/SNF chromatin remodeling complex (2, 3). SWI/SNF is a multi-subunit complex, existing in three main forms, which is involved at several levels of transcriptional regulation (4, 5). Another chromatin modifying complex that interacts with FET fusion oncoproteins is polycomb repressive complex 2 (PRC2), which has opposing roles compared with SWI/SNF, and their interplay is known to be important in gene regulation (6, 7). Approximately 20% of all human cancers carry mutations in genes encoding SWI/SNF components (8). FET sarcomas and leukemias lack mutations in the SWI/SNF and PRC2 coding genes. Instead, the complexes are affected, at least in part, *via* a common molecular mechanism mediated by FET fusion oncoproteins (2).

Myxoid liposarcoma (MLS) is one of the most common types of FET sarcomas and is characterized by the *FUS-DDIT3* or the less common *EWSR1-DDIT3* fusion oncogene (9). The majority of MLS patients are successfully treated, but some tumors develop chemotherapy resistance. In several cancer types, chemotherapy resistance has been linked to subpopulations of tumor cells with stem cell characteristics, commonly referred to as cancer stem cells (CSCs) (10, 11). The JAK-STAT signaling pathway has been associated with the CSC population in several tumor types (12–14). The pathway is activated by binding of ligands to cytokine receptors followed by activation of JAK kinases and STAT proteins that ultimately regulate a set of specific target genes (15). We have previously shown that MLS tumors contain CSC-like cells that can form non-adherent spheres, efflux Hoechst dye, and resist the chemotherapy drug doxorubicin and that all these properties are connected to JAK-STAT signaling (16). In addition, combined doxorubicin treatment and JAK1/2 inhibition resulted in a synergistic effect. However, the molecular connections between the FET fusion oncoprotein FUS-DDIT3 and JAK-STAT signaling remain unknown.

Here, we address this question by analyzing the effects of FUS-DDIT3 expression on STAT3 levels. Next, we determined downstream target genes and networks affected by FUS-DDIT3 expression and JAK1/2 inhibition using RNA sequencing and verified a subset by quantitative PCR. Bioinformatics analysis of published chromatin immunoprecipitation sequencing data sets was used to define FUS-DDIT3- and STAT3-regulated genes. We further studied CD44, an identified downstream target of JAK1/2 inhibition, with molecular and cellular assays. Finally, we studied FUS-DDIT3 interactions with phosphorylated STAT3 (pSTAT3), SWI/SNF, and PRC2 subunits using immunoprecipitation followed by Western blot analysis. Our results suggest that FUS-DDIT3 affects JAK-STAT signaling, which opens up for targeted therapies in MLS.

## Material and Methods

### Cell Culture

The fibrosarcoma cell line HT1080 (available at ATCC, Manassas, VA, USA) (17), either wild-type or stably transfected with either FUS-DDIT3 in the pEGFPN1 vector or the pEGFPN1 vector only (18), and the MLS cell lines 402-91 (19), 2645-94 (20), and 1765-92 (20) were all cultured in complete media, containing RPMI 1640 GlutaMAX supplemented with 5% fetal bovine serum, 100 U/ml penicillin, and 100 µg/ml streptomycin (all Gibco, Thermo Fisher Scientific, Waltham, MA, USA). Cell line identities were controlled by the cell line authentication test performed by Eurofins (Linköping, Sweden). For transfected cells, the media was additionally supplemented with 500 µg/ml geneticin (Gibco, Thermo Fisher Scientific). Cells were kept at 37°C and 5% CO_2_ and passaged using 0.25% trypsin and 0.5 mM EDTA (Gibco, Thermo Fisher Scientific).

For drug treatments, cells were treated for 24 h with 2.5 µM ruxolitinib (Selleckchem, Munich, Germany). For drug titration experiments, 1:2 dilution series, ranging from 3 µM to 5.9 nM, were used.

### Western Blot

Whole-cell extracts were prepared by lysing cells in RIPA lysis buffer supplemented with 1× Halt Protease and Phosphatase Inhibitor Cocktail (both Thermo Scientific, Thermo Fisher Scientific) and scraping cells off the surface. Concentrations were quantified with the DC protein assay (Bio-Rad, Hercules, CA, USA).

Proteins were separated on SDS-PAGE gels using the NuPAGE gel electrophoresis system (Invitrogen, Thermo Fisher Scientific), according to the instructions of the manufacturer. Briefly, samples were mixed with 1× NuPAGE LDS sample buffer and 10% NuPAGE sample reducing agent followed by incubation for 10 min at 70°C. Gel electrophoresis was run on 4%–12% Bis-Tris gels followed by transfer onto polyvinylidene difluoride membranes (Invitrogen, Thermo Fisher Scientific). Blocking of membranes was performed with 5% skim milk (Merck, Darmstadt, Germany) or 5% bovine serum albumin (Sigma-Aldrich, St. Louis, MO, USA) in TBS-T buffer (50 mM Tris–HCl, pH 6.8, 50 mM NaCl, and 0.1% Tween 20; all from Sigma-Aldrich). Antibody incubation was performed overnight at 4°C with primary antibodies against BAF57 (#ab131328, Abcam, Cambridge, UK, diluted 1:1,000), DDIT3 (#15204-1, Proteintech, Rosemont, IL, USA, diluted 1:500), EZH2 (#07-689, Merck Millipore, Merck, diluted 1:1,000), GAPDH (#ab9484, Abcam, diluted 1:1,000 or #60004-1Ig, Proteintech, diluted 1:50,000), GFP (#632381, Clontech, Takara Bio, Kusatsu, Japan, diluted 1:2,000), Histone H4 (#04-858, Merck Millipore, Merck, diluted 1:30,000), STAT3 (#12640, Cell Signaling Technology, Danvers, MA, USA, diluted 1:1,000), or phosphorylated STAT3 (#9131, Cell Signaling Technology, diluted 1:1,000) followed by incubation with anti-mouse (#32430, Invitrogen, Thermo Fisher Scientific, diluted 1:1,000) or anti-rabbit (#32460, Invitrogen, Thermo Fisher Scientific, diluted 1:1,000) horseradish-peroxidase-conjugated secondary antibody for 1 h at room temperature. Detection was performed by incubating membranes with SuperSignal West Dura Extended Duration Substrate or SuperSignal West Femto Maximum Sensitivity Substrate (Thermo Scientific, Thermo Fisher Scientific) and detecting chemiluminescence signals using ImageQuant LAS 4000 mini or ImageQuant Amersham 800 (both GE Healthcare, Chicago, IL, USA). Bands were quantified using ImageJ (21). Statistics was performed using Prism v8.0.0 (GraphPad Software Inc., La Jolla, CA, USA).

### RNA Extraction

Cells were washed with DPBS and then lysed with either RLT lysis buffer (Qiagen, Hilden, Germany) supplemented with β-mercaptoethanol (MP Biomedicals, Santa Ana, CA, USA) or QIAzol lysis reagent (Qiagen) before collection by scraping. RNA was extracted using either RNeasy micro kit for RLT-lysed samples or miRNeasy micro kit for QIAzol-lysed samples (both from Qiagen), according to the instructions of the manufacturer with DNase treatment included. RNA quality was assessed using either Agilent 6000 Nano Kit on a 2100 BioAnalyzer instrument or DNF-471 RNA kit on a Fragment Analyzer (all from Agilent Technologies, Santa Clara, CA, USA), according to the instructions of the manufacturer. Concentrations were quantified with Qubit 4 Fluorometer using the Qubit RNA HS Assay Kit (Invitrogen, Thermo Fisher Scientific). RNA was stored at −80°C.

### RNA Sequencing

Sequencing libraries were prepared according to the Smart-Seq2 protocol (22) with some modifications. Reverse transcription was performed by first adding 1 mM dNTP (Sigma-Aldrich) and 1 µM biotinylated adapter sequence-containing oligo-dT_30_VN (5′-biotin-AAGCAGTGGTATCAACGCAGAGTACT30VN-3′, Sigma-Aldrich or IDT Technologies, Coralville, IA, USA) to 10 ng total RNA for an initial hybridization step at 72°C for 3 min on a T100 instrument (Bio-Rad). Subsequently, 1× first-strand buffer (50 mM Tris–HCl pH 8.3, 75 mM KCl, and 3 mM MgCl_2_), 5 mM dithiothreitol (both Invitrogen, Thermo Fisher Scientific), 10 mM MgCl_2_, 1 M betaine (Sigma-Aldrich), 0.6 µM biotinylated adapter sequence-containing template-switching oligonucleotide (5′-biotin-AAGCAGTGGTATCAACGCAGAGTACATrGrG+G-3′ with rG = riboguanosine and +G = locked nucleic acid modified guanosine, Eurogentec, Liège, Belgium), 15 U RNaseOUT, and 150 U SuperScript II (both Invitrogen, Thermo Fisher Scientific) were added to a 15-µl reaction volume followed by reverse transcription at 42°C for 90 min and 70°C for 15 min in a T100 instrument. All concentrations refer to the final reverse transcription reaction. Complementary DNA was stored at −20°C.

Preamplification was performed by mixing 1× KAPA Hifi HotStart Ready Mix (KAPA Biosystems, Wilmington, MA, USA) and 0.1 µM primer (5′-AAGCAGTGGTATCAACGCAGAGT-3′; Sigma-Aldrich) with 7.5 µl cDNA to a 50-µl reaction volume followed by preamplification at 98°C for 20 s, 67°C for 15 s, and 72°C for 6 min, and a final additional incubation at 72°C for 5 min in a T100 instrument. Samples were frozen on dry ice directly after the final 72°C incubation and stored at −20°C.

Samples were purified using Agencourt AMPure XP (BD Biosciences, San Jose, CA, USA) with a beads-to-sample ratio of 0.8. First, the sample and beads were mixed thoroughly with pipetting followed by 5 min incubation at room temperature on the bench and an additional 5 min incubation on a DynaMag magnet (Thermo Fisher Scientific). Next, the supernatant was discarded and samples were washed twice with 200 µl 80% ethanol (Solveco, Rosersberg, Sweden). After the beads were dried, the samples were eluted using 17.5 µl RNase/DNase-free water (Invitrogen, Thermo Fisher Scientific) followed by 2 min incubation at room temperature on the bench and an additional 2 min incubation on the magnet and 15 µl purified DNA was retrieved. Quality assessment and determination of concentration were performed using either Agilent High Sensitivity DNA Kit (Agilent Technologies) on a 2100 BioAnalyzer Instrument or DNF-474 High Sensitivity NGS kit (Agilent Technologies) on a Fragment Analyzer and 100 pg cDNA was forwarded to tagmentation. Purified cDNA was stored at −20°C.

For library preparation of purified cDNA, the Nextera XT DNA Library Preparation Kit and Nextera XT Index Kit v2 (Illumina, San Diego, CA, USA) were used. Tagmentation was performed by adding 10 µl Tagment DNA Buffer and 5 µl Amplicon Tagment Mix to a 5-µl sample followed by tagmentation at 55°C for 5 min in a T100 instrument. The reaction was stopped by adding 5 µl neutralized Tagment buffer followed by centrifugation for 1 min at 1,100 rpm and 5 min incubation at room temperature. Indexing and library amplification were performed by adding 15 µl Nextera PCR Master Mix and 5 µl of each index i7 and index i5 adapters to a reaction volume of 50 µl followed by amplification at 72°C for 3 min, 95°C for 30 s and 16 cycles of amplification at 95°C for 10 s, 55°C for 30 s, and 72°C for 30 s, and a final additional incubation at 72°C for 5 min in a T100 instrument.

Samples were purified using Agencourt AMPure XP with a beads-to-sample ratio of 0.6 as described above. Concentrations were measured with Qubit 4 Fluorometer using Qubit dsDNA High Sensitivity Assay Kit (Invitrogen, Thermo Fisher Scientific). Quality assessment and size distribution measurement was performed with DNF-474 High Sensitivity NGS kit on a Fragment Analyzer and libraries were pooled to equimolarity. Thereafter, either the pool was forwarded to sequencing performed by the Genomics Core facility at the University of Gothenburg using a NextSeq 500 instrument (Illumina) or the pool was quantified using the NEBNext Library Quantification kit (New England BioLabs, Ipswich, MA, USA) followed by sequencing on a MiniSeq instrument (Illumina) using 1% PhiX control (Illumina) and clustered at 1.8 pM. In both cases, paired-end sequencing with a read length of 2 times 75 bp was used.

After sequencing, alignment was performed using STAR v2.6 (23) with the ENSEMBL GRCh38 reference genome. Read count was performed with HTSeq v0.9.1 (24). Data filtering was performed by excluding genes with a total count number below 10. Differential expression analysis was performed in R v3.6 using DESeq2 (25), based on shrink estimation for dispersion and fold change using a negative binomial distribution model and using Benjamini–Hochberg to calculate adjusted *p*-values. Significantly regulated genes were selected based on adjusted *p*-value ≤0.05 and ≥2-fold regulation. Functional enrichment analysis was performed in R v3.6 using the enricher function from the clusterProfiler package (26). To match the settings used by the molecular signature database (MSigDB) enrichment web tool (27, 28), the background was instead defined by the totality of human genes curated by the HGNC (HUGO Gene Nomenclature Committee) with gene sets retrieved from MSigDB v7.0 or 7.2. A protein network based on regulated genes was created using Cytoscape v3.8.0 (29) with network and interaction data retrieved from STRING (30) using the StringApp v1.5.1 (31), allowing all types of associations included in the protein query function of the StringApp. Values of betweenness centrality were retrieved from the built-in Network Analyzer tool (32). Fisher’s exact test was performed in R v3.6 using the fisher.test function where all genes included in gene mapping using the HGNC-approved nomenclature were applied as the total number of genes.

The RNA sequencing data are available in NCBI’s Gene Expression Omnibus (GEO) database (33) (https://www.ncbi.nlm.nih.gov/geo/) with accession number GSE149650 (34). Note: Some sequenced samples were used elsewhere (2).

### Quantitative Real-Time PCR

Complementary DNA was synthesized using GrandScript cDNA Synthesis Kit (TATAA Biocenter, Gothenburg, Sweden) in 10 µl reactions, containing 1× GrandScript RT reaction mix, 0.5 µl GrandScript RT enzyme (both TATAA Biocenter), RNase/DNase-free water, and 500 ng total RNA. Reverse transcription was performed on a T100 Thermal Cycler at 22°C for 5 min, 42°C for 30 min, 85°C for 5 min, and finally cooling to 4°C. Complementary DNA was diluted 1:10 with RNase/DNase-free water and stored at −20°C before downstream analysis.

Quantitative PCR was performed in 6 µl reactions, containing 1× SYBR GrandMaster Mix (TATAA Biocenter), 400 nM of each primer (Sigma-Aldrich, **Supplementary Table 1**), and 2 µl diluted cDNA. A CFX384 Touch Real-Time PCR System (Bio-Rad) was used with the temperature profile: 95°C for 2 min, 40 cycles of amplification at 95°C for 5 s, 60°C for 20 s, 70°C for 20 s, followed by melting curve analysis: 65°C to 95°C, with an increment of 0.5°C per 5 s. The expected PCR product sizes were confirmed for each primer pair using Fragment Analyzer analysis with the DNF-915 dsDNA Reagent Kit (Agilent Technologies), according to the instruction of the manufacturer. Bio-Rad CFX Maestro 1.1 version 4.1 was used to determine cycles of quantification values by regression (Bio-Rad). Data were preprocessed in GenEx v7.1 (MultiD, Gothenburg, Sweden) and suitable reference genes were identified with NormFinder (35). Data were normalized to the expression of reference genes and then transformed to relative quantities in log2 scale. Quantitative PCR experiments were conducted in accordance with the Minimum Information for Publication of Quantitative Real-Time PCR Experiments guidelines (36).

### Chromatin Immunoprecipitation Sequencing Data Analysis

To retrieve FUS-DDIT3 target genes, we analyzed a published chromatin immunoprecipitation data set from the MLS cell line 402-91 (37), available through the Sequence Read Archive (SRA) database (#SRR6792600) (38). To define common STAT3 target genes, we analyzed chromatin immunoprecipitation sequencing data sets available through the ENCODE database: from the human lymphoblastoid cell line GM12878 (#ENCSR000DZV) (39), the cervical adenocarcinoma cell line HeLa S3 (#ENCSR000EDC) (40), and the non-malignant breast epithelial cell line MCF 10A (#ENCSR000DOZ) (41). Raw reads were mapped to human hg38 reference genome using Burrows-Wheeler Aligner (42), and duplicate reads were identified with picard markDuplicates function and then corrected for in peak calling. Peak calling was performed for each biological replicate in comparison to its input control using Model-based Analysis of ChIP-Seq 2 (MACS2) (43, 44) with peak FDR cutoff = 0.01. Downstream analysis was performed in R v4.1.0 using the BioConductor packages DiffBind and ChIPSeeker. For the STAT3 datasets, the dba.peakset function was used to generate consensus peaks over the two biological replicates with the parameter minOverlap set to 1. Additionally, the dba.venn function was used to determine overlapping peaks between the three cell lines. Peaks were annotated to their neighboring gene with R package TxDb.Hsapiens.UCSC.hg38.knownGene if the peak was within 3,000 bp from the transcription start. Genes that were called in two out of three cell lines were used to generate a common STAT3 target list, which was compared with RNA sequencing data.

### Flow Cytometry Analysis

The protein expression level of CD44 was analyzed using BD Accuri C6 flow cytometer (BD Biosciences). Trypsinized cells (10^5^–10^8^ cells/ml) were suspended in complete medium and stained with 31.3 ng/ml CD44 monoclonal antibody (IM7) PE-Cyanine5 (#15-0441-82, Gibco, Thermo Fisher Scientific) for 30 min at room temperature. Then, cells were washed twice and finally resuspended in Hanks’ balanced salt solution (Gibco, Thermo Fisher Scientific). Cell aggregates were removed by filtering using a 70-µm cell strainer (BD Biosciences) before flow cytometry analysis. Unstained control cells were used to discriminate positively stained cells.

### Immunoprecipitation

For immunoprecipitation experiments, cells were treated with 30 ng/ml leukemia inhibitory factor (Merck) for 30 min at 37°C before nuclear extraction. Cells from three 15-cm cell culture dishes (Nunc, Thermo Fisher Scientific) per cell line were harvested on ice by scraping cells off the surface in DPBS (Gibco, Thermo Fisher Scientific). Cell suspensions were centrifuged for 10 min at 450 rcf and 4°C and resuspended in 500 µl hypotonic lysis buffer (10 mM KCl, 10mM Tris–HCl pH 7.5, and 1.5 mM MgCl_2_; all from Thermo Fisher Scientific) supplemented with 1 mM dithiothreitol (Sigma-Aldrich) and 1× Halt Protease Inhibitor Cocktail (Thermo Scientific, Thermo Fisher Scientific). The added buffer volume is per 100 µl packed cell volume and subsequent buffer volumes are scaled accordingly. Next, cells were incubated for 15 min to swell followed by centrifugation for 5 min at 400 rcf and 4°C. Cells were resuspended in 200 µl hypotonic lysis buffer and cell membranes were disrupted by 5 strokes using a 27-gauge needle (Henke Sass Wolf, VWR, Radnor, PA, USA). Cells were treated for 15 min at 4°C with 5 U/ml Benzonase (Merck) to degrade nucleic acids. Thereafter, the suspension was centrifuged for 20 min at 10,000 rcf and 4°C to separate the cytoplasmic and nuclear fractions. The pellet, containing the nuclear fraction, was resuspended in 67 µl high-salt extraction buffer (0.42 M KCl, 10 mM Tris–HCl pH 7.5, and 0.1 mM EDTA, all from Thermo Fisher Scientific, 10% glycerol, Merck) supplemented with 1× Halt Protease Inhibitor Cocktail, and proteins were extracted by incubation for 30 min in an icebox under gentle agitation. The suspension was centrifuged for 5 min at 20,000 rcf and 4°C. The supernatant containing the nuclear fraction with effective salt concentration of 250 mM KCl was collected and diluted to a physiological level, 150 mM KCl, with dilution buffer (10 mM Tris–HCl pH 7.5, 0.1 mM EDTA, and 10% glycerol), supplemented with 1× Halt Protease Inhibitor Cocktail. For sequential salt extracts, the remaining nuclear pellet was resuspended and agitated for 30 min in an icebox using 67 µl of the same high-salt nuclear extraction buffer but with an even higher salt concentration (0.6 M KCl, 10 mM Tris pH 7.5, 0.1 mM EDTA, and 10% glycerol, supplemented with 1× Halt Protease Inhibitor Cocktail). After collection of this 500 mM KCl fraction, the nuclear pellet was resuspended a third time, now with a 1.2-M KCl high-salt extraction buffer and the corresponding 1,000 mM fraction was collected after 30 min of incubation. Each fraction was collected after centrifugation for 5 min at 20,000 rcf and 4°C and diluted to 150 mM salt concentration before freezing. All nuclear extracts were snap-frozen on dry ice and stored at −80°C.

Immunoprecipitation was performed on nuclear extracts using Dynabeads MyOne streptavidin T1 (Invitrogen, Thermo Fisher Scientific). The nuclear extract (50 µg) was diluted to 250 µl with IP wash buffer (150 mM KCl, 10 mM Tris–HCl pH 7.5, 0.1 mM EDTA, and 10% glycerol) supplemented with 1× Halt Protease Inhibitor Cocktail. Beads were blocked and incubated with antibody for 30 min by incubating 37.5 µl beads per reaction in 400 µl 1× Rotiblock (Carl Roth, Karlsruhe, Germany) in IP wash buffer and 5 µg antibody of either DDIT3-biotin (NB600 1335B, Novus Biologicals, Littleton, CO, USA) or IgG-biotin (sc-2762, Santa Cruz Biotechnology, Santa Cruz, CA, USA). The beads were then washed three times in 400 µl IP wash buffer to remove unbound antibody. The extracts were added to the antibody-bound beads and incubated overnight at 4°C on an end-over-end mixer. After incubation, the supernatant was collected as the non-bound sample, containing proteins not bound to the target. The beads were then washed three times in 400 µl IP wash buffer, each for 5 min on an end-over-end mixer at 4°C. Elution was performed twice using 25 µl 2× NuPAGE LDS sample buffer and 10% NuPAGE sample reducing agent (both from Invitrogen, Thermo Fisher Scientific) for 10 min at 500 rpm and 90°C. Eluates were pooled and samples were stored at −20°C before Western blot analysis. To minimize background signals, low protein-binding tubes (Sarstedt, Nümbrecht, Germany) were used and beads were moved to new tubes before elution.

## Results

### FUS-DDIT3 Expression Activates JAK-STAT Signaling

**Figure 1:**
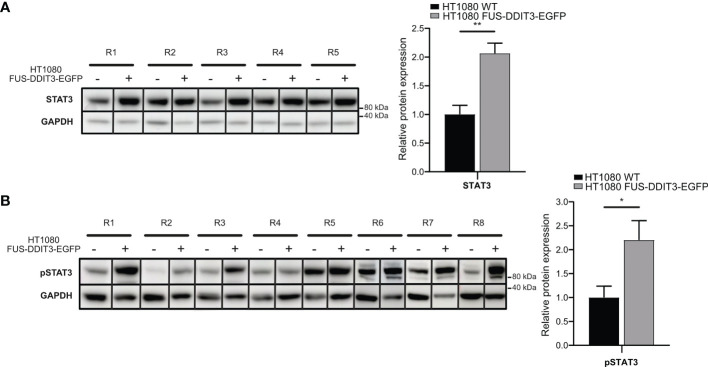
FUS-DDIT3-induced STAT3 expression. **(A)** Western blot analysis of STAT3 using GAPDH as loading control in HT1080 cells with and without FUS-DDIT3 expression [HT1080 FUS-DDIT3-EGFP and HT1080 wild-type (WT), respectively]. Left panel: R1–R5 correspond to samples collected at the same time for both cell lines in different cell passages. Right panel: Relative protein expression of STAT3 normalized to GAPDH. Expression values were mean-centered in relation to HT1080 WT samples. Mean ± SEM is shown, *n* = 5. **p ≤ 0.01, unpaired Student’s *t*-test. Complete Western blot membranes are shown in **Supplementary Figure 2**. **(B)** Western blot analysis of phosphorylated STAT3 (pSTAT3) using GAPDH as loading control in HT1080 cells with and without FUS-DDIT3 expression (HT1080 FUS-DDIT3-EGFP and HT1080 WT, respectively). Left panel: R1–R8 correspond to samples collected at the same time for both cell lines in different cell passages. Right panel: Relative protein expression of pSTAT3 normalized to GAPDH. Expression values were mean-centered between Western blots in relation to HT1080 WT samples. Mean ± SEM is shown, *n* = 8. *p ≤ 0.05, unpaired Student’s *t*-test. Complete Western blot membranes are shown in **Supplementary Figure 2**.

To determine if the MLS-specific fusion oncoprotein FUS-DDIT3 regulates the JAK-STAT pathway, we compared the protein expression of STAT3 in the fibrosarcoma cell line HT1080 with and without ectopic FUS-DDIT3 expression. Western blot analysis showed a significant increase in both STAT3 and pSTAT3 (Tyr705) levels when FUS-DDIT3 was expressed in HT1080 cells (**Figures 1A, B**). The expression levels of pSTAT3 and FUS-DDIT3 in fusion oncogene-expressing HT1080 cells were in the same range as in the three MLS cell lines as shown by Western blot analysis (**Supplementary Figure 1**).

### FUS-DDIT3 Expression and Ruxolitinib Treatment Regulate Overlapping Genes

**Figure 2:**
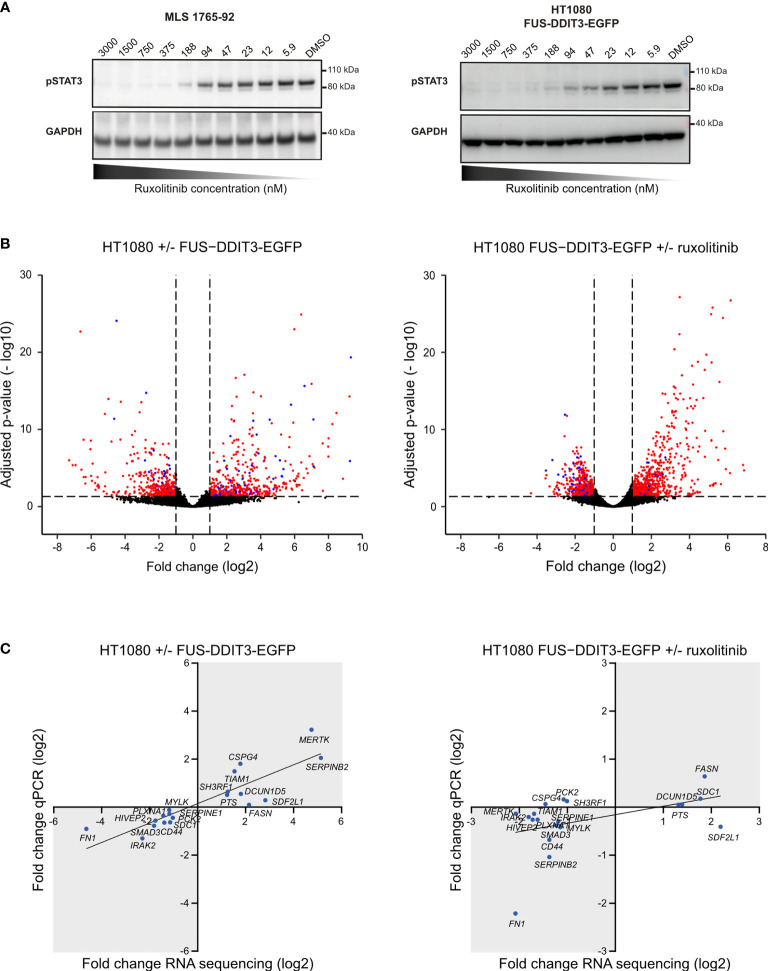
Genes regulated by FUS-DDIT3 expression and ruxolitinib treatment. **(A)** Ruxolitinib dose-dependent phosphorylated STAT3 expression. Western blot analysis of pSTAT3 using GAPDH as loading control in myxoid liposarcoma (MLS) 1765-92 cells and HT1080 FUS-DDIT3-EGFP cells treated for 24 h with 5.9–3,000 nM ruxolitinib. DMSO was used as control, i.e., 0 nM ruxolitinib. Complete Western blot membranes are shown in **Supplementary Figure 2**. **(B)** Differential gene expression analysis. Volcano plots showing significantly regulated genes comparing cells with and without FUS-DDIT3 expression (HT1080-FUS-DDIT3-EGFP vs. HT1080 wild-type) and FUS-DDIT3-expressing HT1080 cells with and without ruxolitinib treatment, respectively. Significantly regulated genes, with fold change ≥ 2 and adjusted *p*-value ≤ 0.05, are shown in either red or blue. Blue dots represent genes that are commonly regulated by both FUS-DDIT3 expression and ruxolitinib treatment. *n* = 3–4 per condition. **(C)** Quantitative PCR validation of RNA sequencing data. The linear fits are shown to guide the eye. Genes in gray areas are considered to be validated. *n* = 4 per condition.

To identify common downstream targets of FUS-DDIT3 expression and JAK1/2 signaling, we performed RNA sequencing, analyzing HT1080 cells with and without FUS-DDIT3 expression as well as HT1080 cells with FUS-DDIT3 expression treated with the JAK1/2 inhibitor ruxolitinib. The inhibition effect of ruxolitinib in HT1080 and MLS cells was shown by dose-dependent downregulation of pSTAT3 (Tyr705) using Western blot analysis (**Figure 2A** and **Supplementary Figure 3**). Differential expression analysis identified 936 significantly regulated genes between HT1080 FUS-DDIT3 and HT1080 wild-type cells (**Figure 2B** and **Supplementary Table 2**). Next, 1,297 significantly regulated genes were identified between ruxolitinib-treated and untreated HT1080 FUS-DDIT3 cells (**Figure 2B** and **Supplementary Table 3**). In both cases, somewhat more genes (54% and 57%, respectively) were upregulated than downregulated. The regulation of 19 randomly selected genes were validated using quantitative PCR where 19 out of 19 genes and 15 out of 19 genes were confirmed to be regulated in the same direction for FUS-DDIT3 expression and ruxolitinib treatment, respectively (**Figure 2C**).

**Figure 3:**
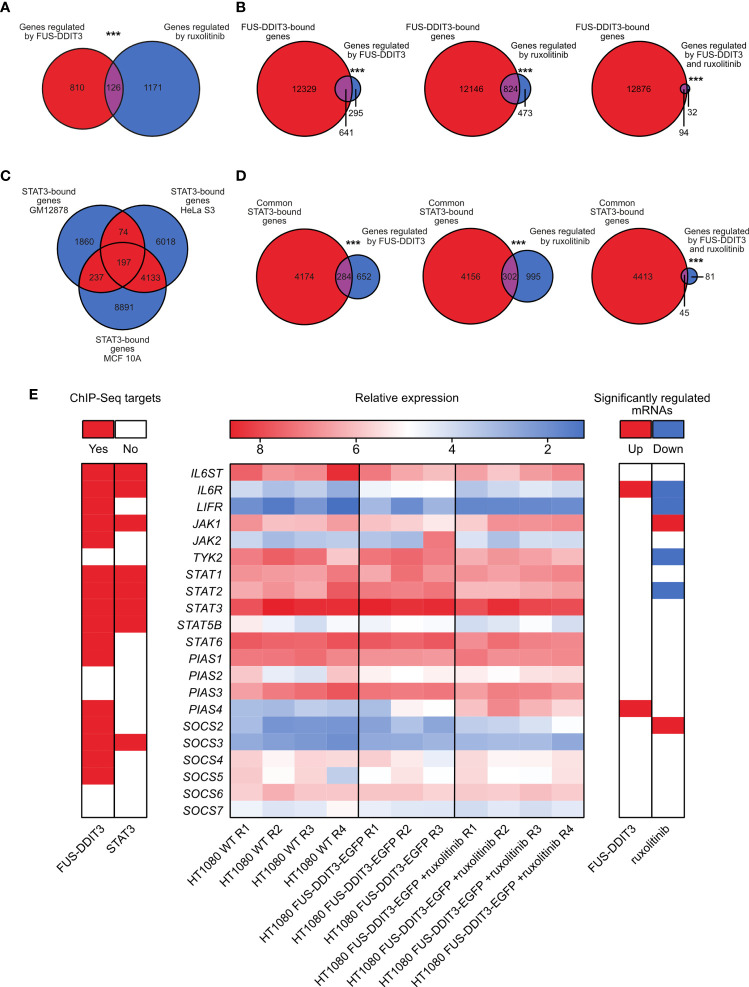
Combined gene expression and chromatin immunoprecipitation sequencing data analysis. **(A)** Venn diagram showing the overlap between genes significantly regulated by FUS-DDIT3 expression and ruxolitinib treatment. ***p ≤ 0.001, Fisher’s exact test. **(B)** Venn diagrams showing overlap between FUS-DDIT3-bound genes based on chromatin immunoprecipitation sequencing data and genes regulated by FUS-DDIT3, ruxolitinib, or both FUS-DDIT3 and ruxolitinib. ***p ≤ 0.001, Fisher’s exact test. **(C)** Venn diagram showing overlap between STAT3-bound genes in the human lymphoblastoid cell line GM12878, the cervical adenocarcinoma cell line HeLa S3, and the non-malignant breast epithelial cell line MCF 10A, based on chromatin immunoprecipitation sequencing data. **(D)** Venn diagrams showing overlap between STAT3-bound genes common for at least two cell lines (red-marked areas in “C”) and genes regulated by FUS-DDIT3, ruxolitinib, or both FUS-DDIT3 and ruxolitinib. ***p ≤ 0.001, Fisher’s exact test. Note that the total number of overlapping genes in “C” (*n* = 4641) and “D” (*n* = 4458) are not identical, since the overlap in “C” was based on pairwise peak comparisons, while the comparisons in “D” were based on gene overlaps. **(E)** Heatmap showing relative mRNA expression of canonical JAK-STAT pathway genes in HT1080 wild-type (WT) and HT1080 FUS-DDIT3-EGFP cells as well as HT1080 FUS-DDIT3-EGFP cells treated with ruxolitinib (middle). R1–R4 correspond to biological replicates collected at different cell passages. Values are transformed with rlog transformation using the DESeq2 differential expression analysis tool. *STAT4*, *STAT5A*, and *SOCS1* were excluded due to low expressions in all samples. Significantly up- (red) or downregulated (blue) genes due to FUS-DDIT3 expression or ruxolitinib treatment are shown (right) using fold change ≥ 2 and adjusted *p*-value ≤ 0.05. Confirmed FUS-DDIT3 and STAT3 targets from public chromatin immunoprecipitation data are marked in red (left). ChIP-Seq, chromatin immunoprecipitation sequencing.

Out of the differentially expressed genes, 126 were commonly regulated by FUS-DDIT3 expression and ruxolitinib treatment (**Figure 3A**, **Supplementary Figure 4**, and **Supplementary Table 4**). The number of genes regulated by FUS-DDIT3 was also significantly overrepresented among the genes regulated by ruxolitinib treatment (*p* < 0.001, Fisher’s exact test), indicating overlapping downstream functions for FUS-DDIT3 and JAK-STAT signaling. To study the role of FUS-DDIT3 in gene regulation, we analyzed a publicly available chromatin immunoprecipitation dataset of FUS-DDIT3 target genes in MLS 402-91 cells (**Supplementary Table 5**). The majority of the genes regulated by FUS-DDIT3 expression and ruxolitinib treatment, alone and in combination, were identified as FUS-DDIT3 targets, including 94 of the 126 commonly regulated genes (**Figure 3B**). In all cases, the regulated genes were significantly enriched among the FUS-DDIT3 targets (*p* < 0.001, Fisher’s exact test).

### FUS-DDIT3 Expression and Ruxolitinib Treatment Act Through STAT3

FUS-DDIT3 expression and JAK1/2 inhibition by ruxolitinib may mediate their regulatory effects through other transcription factors than pSTAT3. To determine the importance of STAT3, we analyzed publicly available chromatin immunoprecipitation sequencing data and identified 4,458 genes that STAT3 binds to in at least two out of the three evaluated cell types (**Figure 3C** and **Supplementary Table 6**). Genes regulated by FUS-DDIT3 expression and ruxolitinib treatment, alone and in combination, were significantly enriched among the common STAT3 targets (*p* < 0.001, Fisher’s exact test, **Figure 3D**). More specifically, 45 of the 126 genes commonly regulated by FUS-DDIT3 and ruxolitinib were confirmed STAT3 targets (**Supplementary Table 6**). Of these, 38 were also FUS-DDIT3 targets. These data demonstrate that STAT3 acts as a signaling mediator for FUS-DDIT3 and JAK1/2 signaling in our experimental system.

To investigate the direct effects of FUS-DDIT3 expression and ruxolitinib treatment on JAK-STAT gene regulation, we mapped the mRNA levels of canonical JAK-STAT members (**Figure 3E**). FUS-DDIT3 expression resulted in the upregulation of *IL6R* and *PIAS4*, while ruxolitinib treatment caused the downregulation of *IL6R*, *LIFR*, *TYK2*, and *STAT2* and the upregulation of *JAK2* and *SOCS2*. Chromatin immunoprecipitation sequencing data showed that most JAK-STAT genes are potential FUS-DDIT3 targets, while only a subset is STAT3 targets.

### FUS-DDIT3 Expression and Ruxolitinib Treatment Regulate Overlapping Cellular Properties

**Figure 4:**
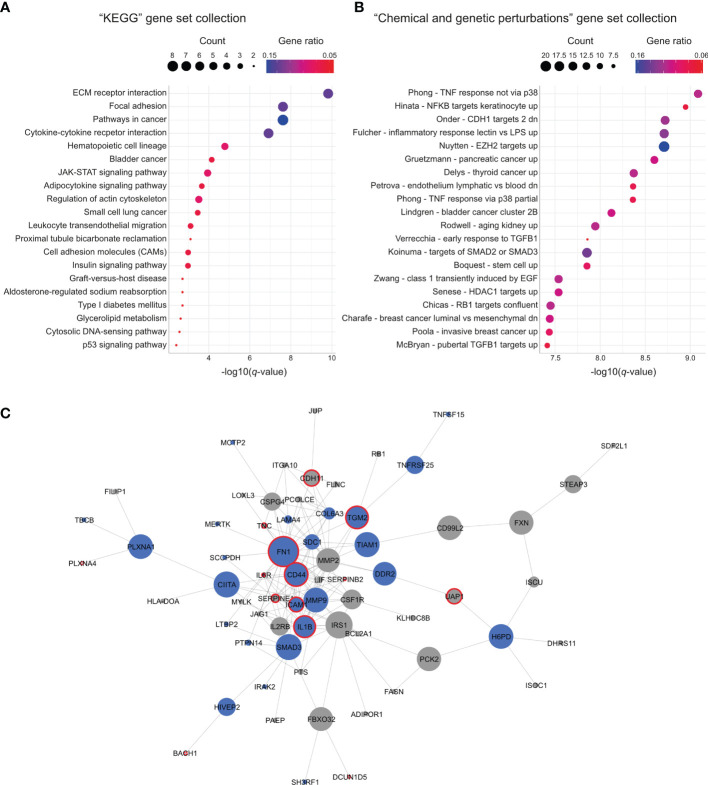
Gene sets and network regulated by both FUS-DDIT3 expression and ruxolitinib treatment. **(A)** Significantly enriched gene sets from the “KEGG” gene set collection using the 126 commonly regulated genes. Top 20 gene sets based on *q*-value are shown. Gene count is indicated by dot size and gene ratio by color. **(B)** Significantly enriched gene sets from the “chemical and genetic perturbations” gene set collection using the 126 commonly regulated genes. Top 20 gene sets based on *q*-value are shown. Gene count is indicated by dot size and gene ratio by color. **(C)** Interaction network created with Cytoscape (29) based on protein interaction data retrieved from STRING (30). Node size is based on betweenness centrality. Nodes marked in blue indicate common STAT3-bound genes and red borders indicate proteins that match to the “Nuytten - EZH2 targets up” gene set from the “chemical and genetic perturbations” gene set collection.

To characterize the 126 genes commonly regulated by FUS-DDIT3 expression and ruxolitinib treatment, we performed functional enrichment analysis. When comparing our gene list with the “KEGG” gene set collection from the molecular signature database (27, 28), we identified several significantly enriched gene sets (**Figure 4A** and **Supplementary Table 7**). The identified gene sets included “adipocytokine signaling pathway” and cytokine signaling, such as the “JAK-STAT signaling pathway.” Several gene sets were also related to extracellular matrix interactions, such as “ECM receptor interaction” and “focal adhesion.” Additionally, functional enrichment analysis with the “chemical and genetic perturbations” collection identified chromatin modification-associated gene sets, i.e., “Nuytten - EZH2 targets up” and “Senese - HDAC1 targets up”, as well as “Boquest - stem cell up,” among others (**Figure 4B** and **Supplementary Table 7**).

To identify the connection between the 126 genes in more detail, we performed network analysis based on known and predicted protein interactions and associations. Out of the 126 genes, 66 genes (52%) were connected in a shared network (**Figure 4C**). In addition, 29 and 53 of the 66 genes were confirmed to be STAT3 and FUS-DDIT3 targets, respectively (**Figure 4C** and **Supplementary Figure 5**), defined by the chromatin immunoprecipitation sequencing data analyses. Central proteins within the network include FN1, CD44, IRS1, and MMP9. Interestingly, several of the central proteins were associated with the PRC2 component EZH2 by matching the “Nuytten - EZH2 targets up” gene set (**Figure 4C**).

### CD44 Is a Target of JAK-STAT Signaling and a Marker of Cancer Stem Cell Properties in Myxoid Liposarcoma

**Figure 5:**
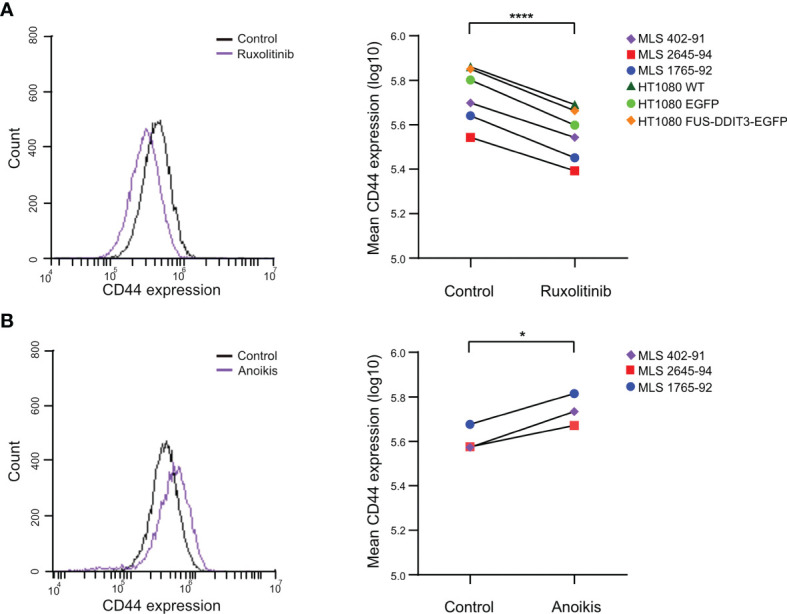
CD44 protein expression analyzed by flow cytometry. **(A)** CD44 regulation by ruxolitinib treatment. The CD44 expression profiles of ruxolitinib-treated myxoid liposarcoma (MLS) 402-91 cells compared with control cells treated with DMSO are shown as an example (left panel). Mean CD44 expressions in ruxolitinib-treated MLS (402-91, 2645-94, and 1765-92) and fibrosarcoma [HT1080 wild-type (WT), HT1080 EGFP, and HT1080 FUS-DDIT3-EGFP] cells are shown (right panel). ****p < 0.0001, paired Student’s *t*-test. **(B)** CD44 regulation by anoikis resistance. The CD44 expression profiles of anoikis-resistant MLS 402-91 cells in comparison to control cell cultures in monolayer are shown as example (left panel). Mean CD44 expressions in anoikis-resistant cells compared with control cells for MLS (402-91, 2645-94, and 1765-92) cells are shown (right panel). *p < 0.05, paired Student’s *t*-test.

CD44, a potential CSC marker (45, 46), was regulated by both FUS-DDIT3 expression and ruxolitinib treatment at the RNA level. To examine whether the CD44 regulation was translated to the protein level, we analyzed its protein levels by flow cytometry in HT1080 cells. To evaluate the relevance of CD44 in MLS, we also analyzed its expression in three MLS cell lines: 402-91, 2645-94, and 1765-92. All cell lines expressed CD44 and inhibition of the JAK-STAT pathway with ruxolitinib treatment caused downregulation in all cell lines (**Figure 5A**). The differences in CD44 expression between HT1080 cells with and without ectopic FUS-DDIT3 expression were negligible. To evaluate if CD44 expression is related to CSC properties in MLS cells, we quantified CD44 expression in anoikis-resistant cells, a property associated with CSCs (47) (**Figure 5B**). Here, our data showed 45%, 25%, and 38% higher CD44 expression in anoikis-resistant MLS 402-91, 2645-94, and 1765-92 cells, respectively.

### FUS-DDIT3 Interacts With Phosphorylated STAT3, SWI/SNF, and PRC2

**Figure 6:**
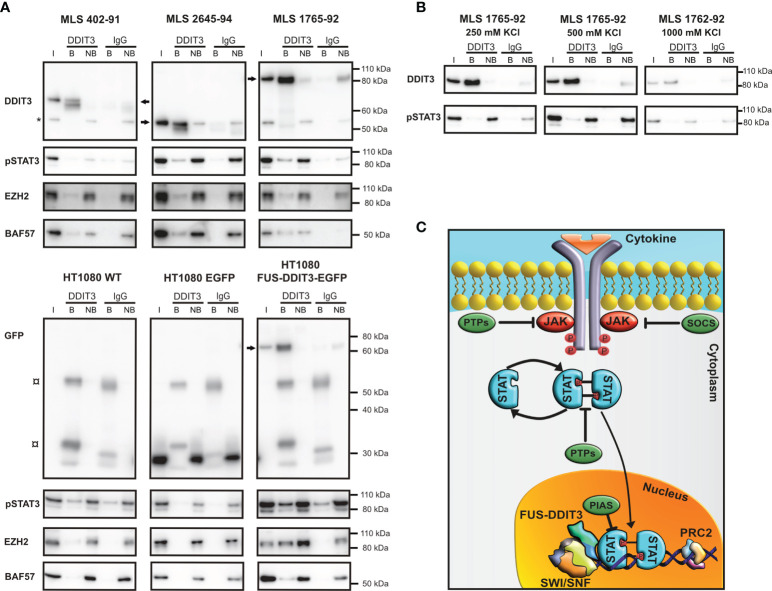
FUS-DDIT3 interacting partners. **(A)** Western blot analysis of DDIT3 immunoprecipitation experiment in myxoid liposarcoma (MLS) (402-91, 2645-94, and 1765-92) and fibrosarcoma [HT1080 wild-type (WT), HT1080 EGFP, and HT1080 FUS-DDIT3-EGFP] cell lines, visualizing FUS-DDIT3 and coimmunoprecipitation of pSTAT3 (Tyr705), EZH2 (PRC2 component), and BAF57 (SWI/SNF component). Cells were treated with leukemia inhibitory factor for 30 min before harvest to maximize the level of pSTAT3. FUS-DDIT3 is detected by a DDIT3 antibody in MLS and a GFP antibody in fibrosarcoma cells. The molecular weights of FUS-DDIT3 variants are different between the cell lines, indicated by arrows. * indicates unspecific background around 55 kDa in input and non-bound samples. ¤ indicates background from secondary antibody interaction with heavy (55 kDa) and light chain (25 and 35 kDa) of immunoprecipitation antibody. **(B)** Western blot analysis of DDIT3 immunoprecipitated in sequential salt extracts (250, 500, and 1,000 mM KCl) in MLS 1765-92 cells, visualizing FUS-DDIT3 and coimmunoprecipitation of pSTAT3. **(C)** Schematic picture of the JAK-STAT signaling pathway. Janus kinases are phosphorylated and activated by cytokine binding to receptors which result in phosphorylation and activation of STAT proteins that translocate into the nucleus, where they can regulate transcription of target genes. The SWI/SNF and PRC2 complexes are potentially involved in the pathway through interactions with FUS-DDIT3 and phosphorylated STAT3 (pSTAT3). Members of the PTP, SOCS, and PIAS families act as inhibitors of the pathway. Non-specific IgG was used as a negative control. I, input sample from the nuclear extract (3% of input); B, bound fraction (proteins bound to target during immunoprecipitation); NB, non-bound fraction (proteins not bound to target during immunoprecipitation). All immunoprecipitation samples were evaluated on the same gel and bands for each antibody were treated equally even though separate rectangles are shown for improved visualization. Complete Western blot membranes are shown in **Supplementary Figure 2**.

To further define the connection between FUS-DDIT3 and JAK-STAT signaling, we performed immunoprecipitation using a DDIT3 antibody targeting only FUS-DDIT3, since no endogenous DDIT3 is expressed in these cells without stress induction. FUS-DDIT3 interacted with pSTAT3 (Tyr705) in nuclear extracts of three MLS cell lines as well as in HT1080 with ectopic expression of FUS-DDIT3 (**Figure 6A**). The strength of the interaction between FUS-DDIT3 and pSTAT3 was evaluated by precipitating FUS-DDIT3 in nuclear extracts with increasing salt concentrations, i.e., sequential salt extraction. The protein binding between FUS-DDIT3 and pSTAT3 remained at 1,000 mM KCl, indicating a strong interaction (**Figure 6B**). Additionally, BAF57 (also known as SMARCE1), a core component of SWI/SNF, and EZH2, the enzymatic component of PRC2, were also present in the precipitated fractions.

## Discussion

Myxoid liposarcoma tumors are genetically stable and contain few mutations, which indicate that FUS-DDIT3 impacts on crucial mechanisms in tumor development, including an instructing master activity that direct tumor cell differentiation (18). JAK-STAT signaling is active in MLS cells and regulates CSC properties, such as chemotherapy resistance (16). These observations prompted us to investigate whether FUS-DDIT3 expression is linked to JAK-STAT signaling. Indeed, we demonstrated that the levels of STAT3 and pSTAT3 were increased in human fibrosarcoma cells with ectopic FUS-DDIT3 expression. At the mRNA level, more canonical JAK-STAT genes were regulated by ruxolitinib treatment compared with FUS-DDIT3 expression, potentially as compensatory effects of JAK1/2 inhibition. However, most members of the JAK-STAT pathway were not regulated at the mRNA level, indicating posttranscriptional JAK-STAT regulation. The chromatin immunoprecipitation data showed that a significant number of regulated genes were FUS-DDIT3 and STAT3 targets. The proportion of FUS-DDIT3 target genes was higher than for STAT3. However, our comparisons of chromatin immunoprecipitation data are not directly comparable, since they are performed in different cell lines and transcription factor target binding is known, at least partly, to be cell-type specific. This can potentially explain the fact that *LIFR*, *TYK2*, and *SOCS2* were not confirmed STAT3 targets despite being significantly regulated by ruxolitinib in our cells. In summary, our data indicate that STAT3 is a key mediator of both FUS-DDIT3 and JAK1/2 signaling. However, other signaling pathways are also potentially involved in regulating the effect seen upon FUS-DDIT3 expression and ruxolitinib treatment. These include PI3K/Akt (48) and YAP1/Hippo (49) for FUS-DDIT3 expression as well as PI3K/Akt (50, 51), ERK2/MAPK (52), and other STATs than STAT3 (15, 53) for JAK1/2 signaling. Further studies are needed to define these signaling pathways and their interplay with STAT3.

We identified 126 genes that were regulated by both FUS-DDIT3 expression and ruxolitinib treatment pointing toward common activation of downstream biological processes. When further characterizing these genes, we identified a protein interaction network where about 80% and more than 40% of the genes also were FUS-DDIT3 and STAT3 targets, respectively. Central proteins within this network included CD44. The glycoprotein CD44 is expressed on several cell types, including embryonic stem cells, and is regarded as a CSC marker (46, 54). Additionally, CD44 has been linked to CSC properties potentially regulated through PDGFRB/STAT3 signaling (55). Here, we confirmed that CD44 is a downstream target of ruxolitinib treatment and a potential CSC marker in MLS. Chromatin immunoprecipitation sequencing data from other cell types confirmed CD44 as a direct STAT3 target gene. The role of CD44 as a CSC marker in sarcomas has also been suggested by others (45, 56). Interestingly, CD44 has been shown to interact with additional proteins that were central in our network. For example, MMP9, a matrix metalloproteinase, is involved in the degradation of the extracellular matrix and connected to tumor cell invasion and metastasis (57). Through this interaction, CD44 potentially mediates tumor cell invasion (58). Additionally, CD44 binds several extracellular matrix proteins, including fibronectin (FN1) (54), an important component of the extracellular matrix that is involved in several processes such as cell adhesion and migration (59). We showed that *CD44*, *FN1*, and *MMP9* were downregulated after both FUS-DDIT3 expression and ruxolitinib treatment, and decreased expression of CD44 (60), MMP9 (61), and FN1 (62) has also been observed in other tumor types after JAK-STAT inhibition. Insulin receptor substrate 1 (IRS1) is another central protein in the network that showed decreased mRNA expression due to FUS-DDIT3 expression and ruxolitinib treatment. IRS1 mediates signaling from various receptors and thereby takes part in several signaling pathways, connected to different cellular processes such as metabolism, cellular growth, and differentiation. It is also known to mediate signaling by JAK activation (63, 64).

In the functional enrichment analysis, we identified several gene sets, such as “JAK-STAT signaling pathway,” “cytokine-cytokine receptor interaction pathway,” and “Boquest – stem cell up” that were expected based on their functional roles in relation to ruxolitinib treatment (16). In addition, “adipocytokine signaling pathway” was interesting due to the role of adipogenesis in MLS, where FUS-DDIT3 has been shown to induce adipogenesis but block complete adipocytic differentiation (18, 65). Interestingly, STAT3 is also involved in early adipogenesis (66, 67), indicating that FUS-DDIT3 and JAK-STAT signaling commonly regulate this MLS-specific feature. As an example, we observed that FN1 was downregulated, which is directly linked to its involvement in adipocytic differentiation (68, 69). Furthermore, we identified gene sets related to chromatin modifiers HDAC1 and EZH2. These gene sets included genes upregulated in osteosarcoma cells after knockdown of histone deacetylase HDAC1 (70) as well as genes upregulated in prostate cancer cells after knockdown of PRC2 component EZH2 (71). Most of the EZH2 target genes were downregulated after ruxolitinib treatment, whereas FUS-DDIT3 expression caused a more complex pattern with both up- and downregulated genes. Several of the genes that overlapped with the EZH2 targets were also central proteins in our interaction network, further indicating that chromatin remodeling may be central among the mechanisms associated with both FUS-DDIT3 expression and JAK-STAT signaling.

Previous studies have revealed a role of chromatin remodeling in MLS through interactions between FUS-DDIT3 and components of SWI/SNF and PRC2 (2, 72). Interestingly, the expression of FUS-DDIT3 in fibrosarcoma cells resulted in significantly increased H3K27me3 levels (2), a process known to be mediated by EZH2 (73). Besides their connection to FUS-DDIT3, both SWI/SNF and PRC2 are also potential mediators of pSTAT3 signaling (74–77). The interaction between pSTAT3 and the SWI/SNF component BRG1 has been confirmed in MLS cells. In addition, BRG1 knockdown led to reduced pSTAT3 levels and reduced number of cells with CSC-like properties, indicating a role for the SWI/SNF complex in JAK-STAT signaling and downstream effects in MLS (16). The PRC2 complex is also involved in JAK/STAT signaling at several levels. For example, JAK2 can mediate EZH2 degradation (78) and EZH2 may enhance STAT3 activation (77, 79). These data prompted us to investigate whether also FUS-DDIT3 and pSTAT3 interacted in MLS cells and we could demonstrate an interaction that remained at 1,000 mM salt concentration. As expected, SWI/SNF subunit BAF57 and PRC2 component EZH2 also precipitated with FUS-DDIT3, but further experiments are needed to determine the exact nature behind these interactions, including which of these are direct binding partners. Our results together with previous studies suggest that FUS-DDIT3 affects the activity of pSTAT3 through SWI/SNF, PRC2, or their antagonistic activity (**Figure 6C**). In addition, their interaction in nuclear extracts together with the FUS-DDIT3 and STAT3 target sites identified in the chromatin immunoprecipitation sequencing data provides an explanation for the many shared target genes. However, further experiments are needed to determine how FUS-DDIT3 affects JAK-STAT signaling mechanistically.

Most MLS patients are successfully treated with combinations of surgery, radiotherapy, and chemotherapy, but some cases remain a clinical problem. Our previous study (16) and data from other tumor entities (80–82) suggest that JAK-STAT inhibition combined with chemotherapy may address this issue by targeting both chemotherapy-resistant cells and proliferating cells. Interestingly, trabectedin has now been implemented in the clinic for the treatment of MLS patients that fail to respond to standard chemotherapy (83). Apart from cytotoxic and antiproliferative effects, trabectedin affects several other processes. In MLS, it particularly promotes adipocytic differentiation through inhibiting FUS-DDIT3 binding to specific promoters (84–86). It would be interesting to evaluate the effect of JAK-STAT inhibition in relation to trabectedin treatment. In this study, we have demonstrated that expression of the MLS-specific fusion oncoprotein FUS-DDIT3 results in increased activation of the JAK-STAT pathway and that FUS-DDIT3 expression and JAK-STAT signaling affect overlapping downstream genes, of which CD44 was connected to cancer stem cell properties in MLS cells. Future *in-vivo* studies are needed to determine the clinical value of JAK-STAT inhibition in MLS, especially in combination with other therapies.

## Data Availability Statement

The datasets presented in this study can be found in online repositories. The names of the repository/repositories and accession number(s) can be found in the article.

## Author Contributions

SD and EJ designed the study, performed the experiments, analyzed the data, and wrote the manuscript. LA performed the experiments and analyzed the data. MLS analyzed the data. ML performed the experiments and analyzed the data. TÖ analyzed the data. PÅ supervised and analyzed the data. AS designed the study, supervised and analyzed the data, and wrote the manuscript. All authors have read and agreed to the published version of the manuscript.

## Funding

This research was funded by Assar Gabrielssons Research Foundation; Johan Jansson Foundation for Cancer Research; Region Västra Götaland, Sweden; Stiftelsen Sigurd och Elsa Goljes Minne; Swedish Cancer Society (19-0306 and 20-1098); Swedish Research Council (2020-01008); Swedish Childhood Cancer Foundation (MTI2019-0008 and 2020-0007); the Swedish state under the agreement between the Swedish government and the county councils, the ALF-agreement (72211, 716321, and 965065); Sweden’s Innovation Agency; The Sjöberg Foundation; and Wilhelm and Martina Lundgren Foundation for Scientific Research.

## Conflict of Interest

AS declares stock ownership and is also a board member in Tulebovaasta, SiMSen Diagnostics, and Iscaff Pharma.

The remaining authors declare that the research was conducted in the absence of any commercial or financial relationships that could be construed as a potential conflict of interest.

## Publisher’s Note

All claims expressed in this article are solely those of the authors and do not necessarily represent those of their affiliated organizations, or those of the publisher, the editors and the reviewers. Any product that may be evaluated in this article, or claim that may be made by its manufacturer, is not guaranteed or endorsed by the publisher.
