# Supplementary material for: FUS-DDIT3 Fusion Oncoprotein Expression Affects JAK-STAT Signaling in Myxoid Liposarcoma
Source: Front Oncol. 2022 Feb 3;12:816894. doi: 10.3389/fonc.2022.816894 (PMC8851354; doi:10.3389/fonc.2022.816894)
Supplement: Supplementary file 1 [file DataSheet_1.pdf]

Supplementary Material

Supplementary Figures

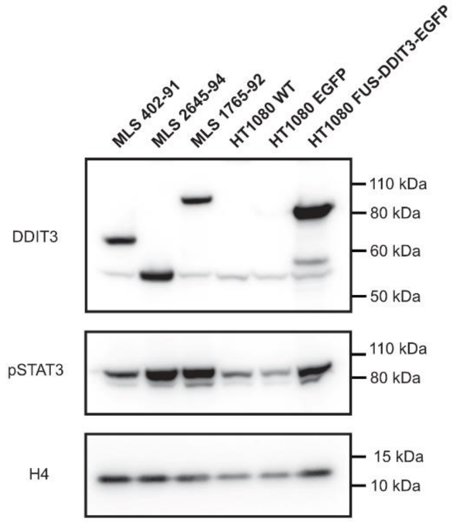

Supplementary Figure 1. FUS-DDIT3 and phosphorylated STAT3 expression in myxoid liposarcoma and fibrosarcoma cells. Western blot analysis of FUS-DDIT3 (DDIT3), phosphorylated STAT3 (pSTAT3, Tyr705) and Histone H4 (H4) in nuclear extracts of myxoid liposarcoma (402-91, 2645-94 and 1765-92) and fibrosarcoma (HT1080 Wild-Type (WT), HT1080 EGFP and HT1080 FUS-DDIT3-EGFP) cells are shown. Histone H4 was used as loading control. Cells had been pretreated with leukemia inhibitory factor. Complete western blot membranes are shown in Supplementary Figure 2.

A

Original WB membranes for Figure 1A

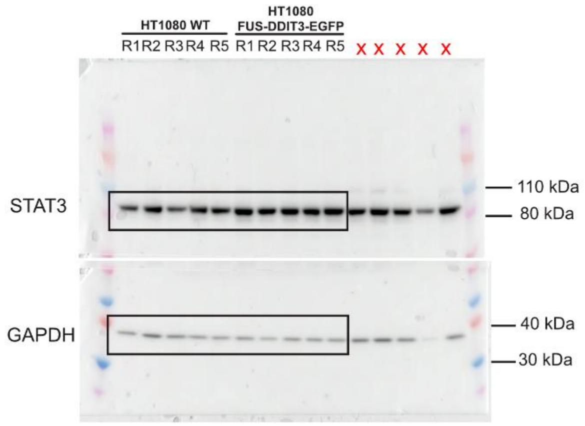

B

Original WB membranes for Figure 1B

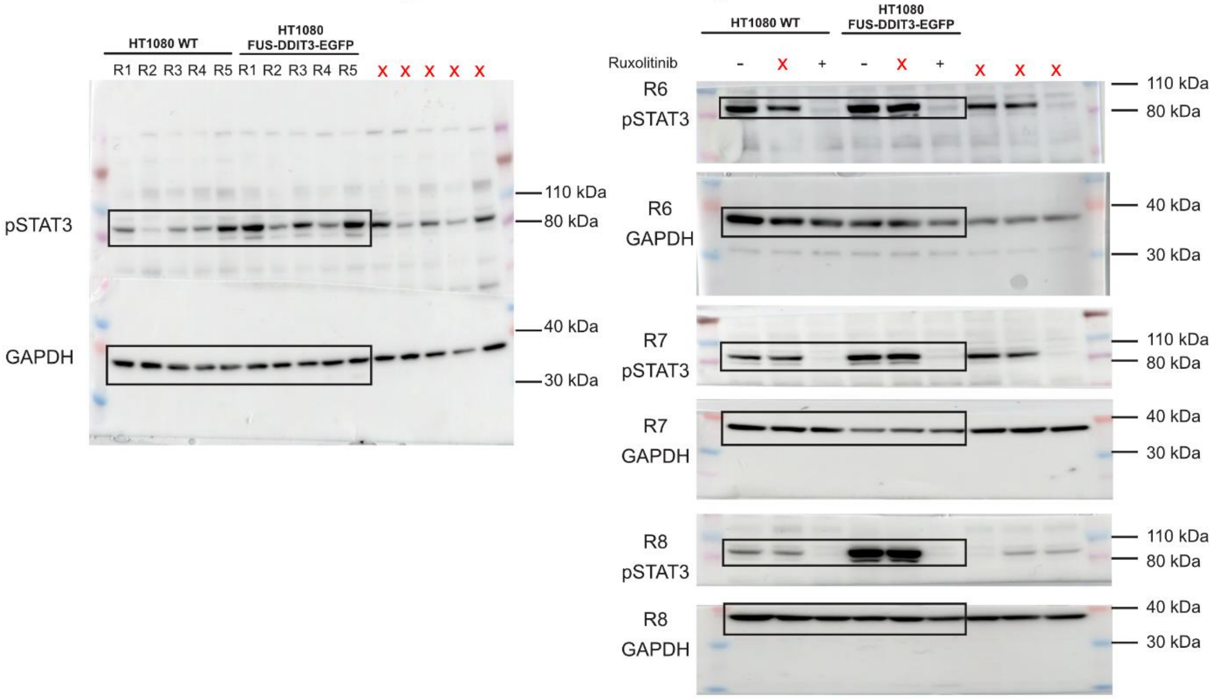

C

Original WB membranes for Supplementary Figure 1

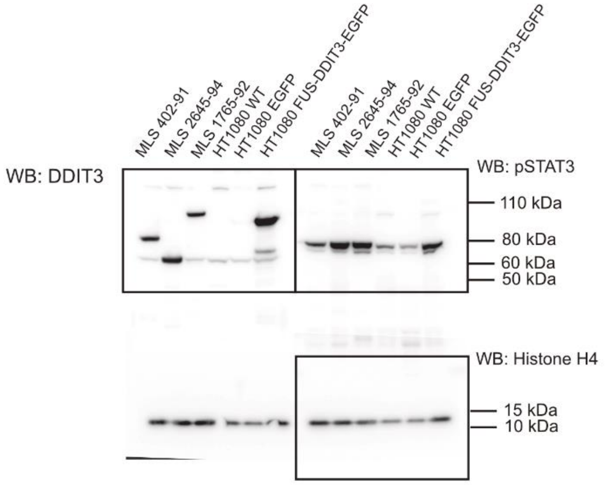

D

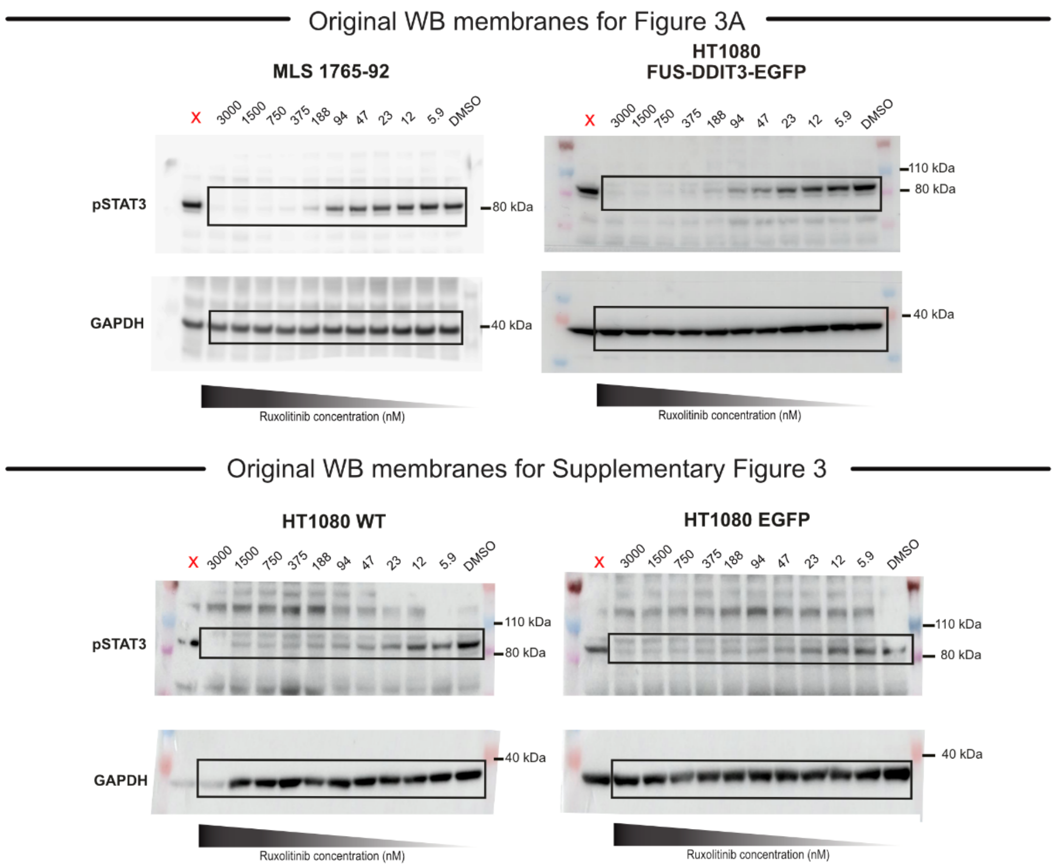
Original WB membranes for Figure 3A
MLS 1765-92
HT1080
FUS-DDIT3-EGFP
X
3000
1500
750
375
188
94
47
23
12
5.9
DMSO
pSTAT3
GAPDH
110 kDa
80 kDa
40 kDa
Ruxolitinib concentration (nM)
Original WB membranes for Supplementary Figure 3
HT1080 WT
HT1080 EGFP

E

Original WB membranes for Figure 5A

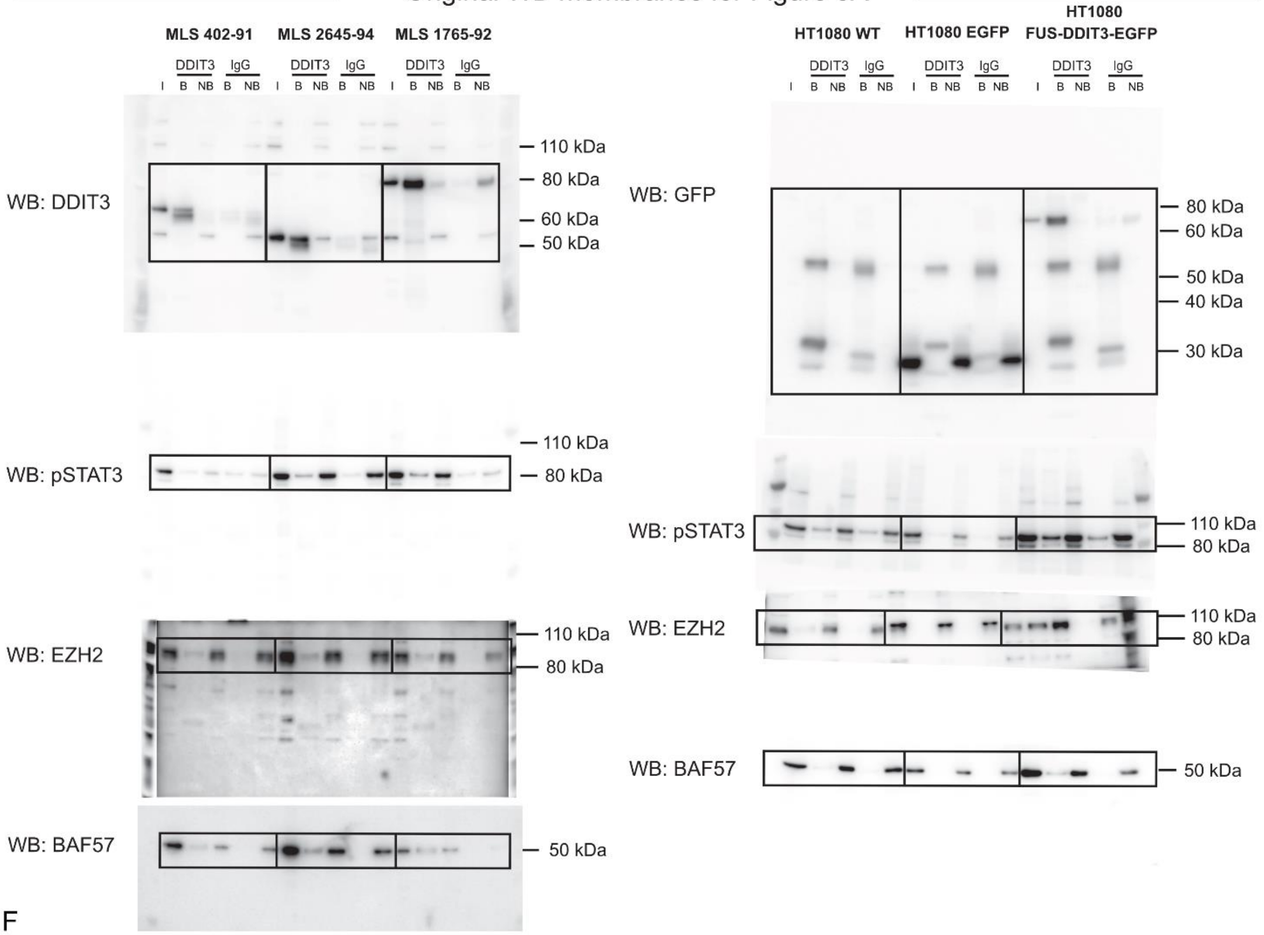

F

Original WB membranes for Figure 5B

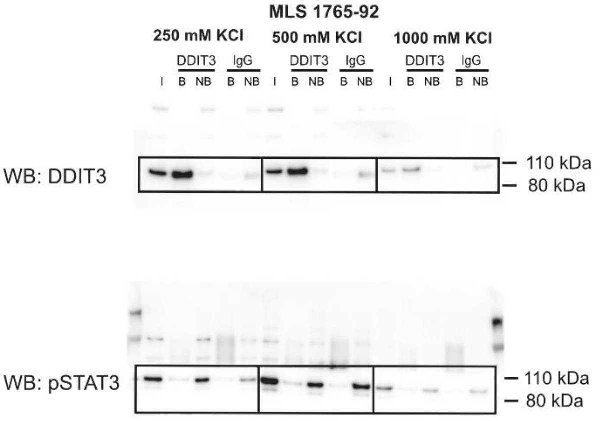

Supplementary Figure 2. Complete western blot membranes. **(A)** FUS-DDIT3-induced STAT3 expression. Original western blot membranes of STAT3 and GAPDH in fibrosarcoma (HT1080 WT and HT1080 FUS-DDIT-EGFP) cells displayed in Figure 1A. **(B)** FUS-DDIT3-induced pSTAT3 expression. Original western blot membranes of pSTAT3 (Tyr705) and GAPDH in fibrosarcoma (HT1080 WT and HT1080 FUS-DDIT-EGFP) cells displayed in Figure 1B. **(C)** Original western blot membranes of FUS-DDIT3, pSTAT3 (Tyr705) and Histone H4 in myxoid liposarcoma (402-91, 2645-94 and 1765-92) and fibrosarcoma (HT1080 WT, HT1080 EGFP and HT1080 FUS-DDIT-EGFP) cells displayed in Figure S1. **(D)** Original western blot membranes of pSTAT3 (Tyr705) and GAPDH in MLS 1765-92 and fibrosarcoma (HT1080 WT, HT1080 EGFP and HT1080 FUS-DDIT-EGFP) cells after treatment with 5.9 - 3000 nM ruxolitinib and DMSO as control displayed in Figures 2A and S3. **(E)** FUS-DDIT3 interaction partners. Original western blot membranes of FUS-DDIT3 immunoprecipitation experiment in myxoid liposarcoma (402-91, 2645-94 and 1765-92) and fibrosarcoma (HT1080 Wild-Type (WT), HT1080 EGFP and HT1080 FUS-DDIT-EGFP) cells, visualizing coimmunoprecipitation of FUS-DDIT3, phosphorylated STAT3 (pSTAT3, Tyr705), EZH2 and BAF57 displayed in Figure 5A. FUS-DDIT3 is detected by a DDIT3 antibody in myxoid liposarcoma cells and a GFP antibody in fibrosarcoma cells. **(F)** Original western blot membranes of FUS-DDIT3 immunoprecipitated in sequential salt extracts (250, 500 and 1000 mM KCl) in MLS 1765-92 cells, visualizing FUS-DDIT3 and coimmunoprecipitation of pSTAT3 (Tyr705) displayed in Figure 5B.

X represents samples that are analyzed on the same western blots, but not relevant for this study.

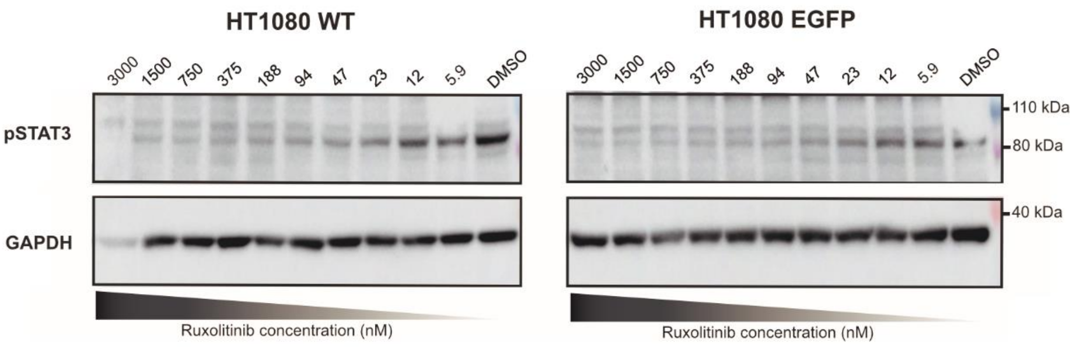

Supplementary Figure 3. Ruxolitinib dose-dependent phoshorylated STAT3 expression. Western blot analysis of phosphorylated STAT3 (pSTAT3, Tyr705) using GAPDH as loading control in HT1080 Wild-Type (WT) and HT1080 EGFP cells treated for 24 hours with 5.9 - 3000 nM ruxolitinib and DMSO as control, *i.e.*, 0 nM ruxolitinib. Complete western blot membranes are shown in Supplementary Figure 2.

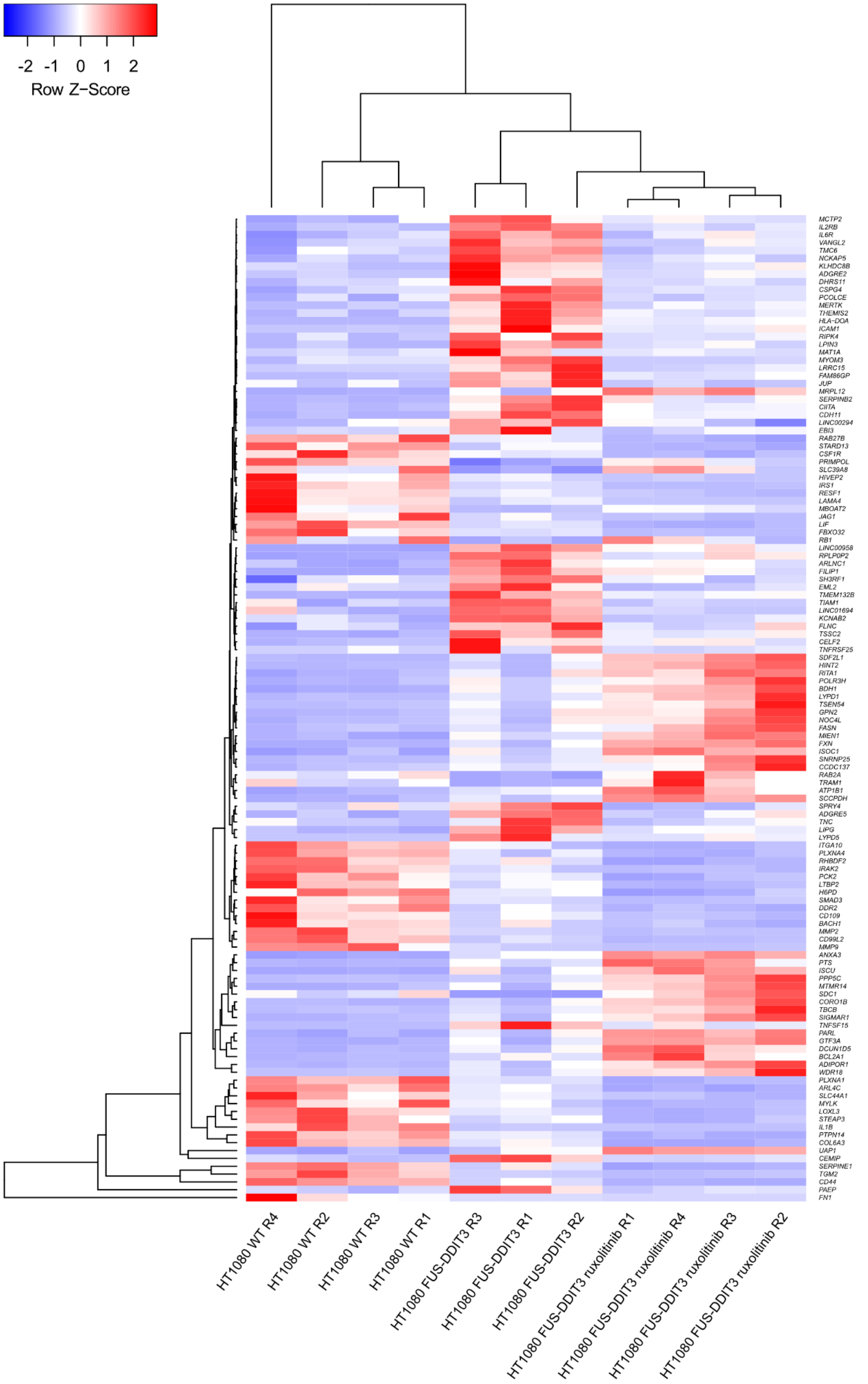

-2 -1 0 1 2
Row Z−Score
MCTP2
IL2RB
IL6R
VANGL2
TMC6
NCKAP5
KLHDC8B
ADGRE2
DHRS11
CSPG4
PCOLCE
MERTK
THEMIS2
HLA−DOA
ICAM1
RIPK4
LPIN3
MAT1A
MYOM3
LRRC15
FAM86GP
JUP
MRPL12
SERPINB2
CIITA
CDH11
LINC00294
EBI3
RAB27B
STARD13
CSF1R
PRIMPOL
SLC39A8
HIVEP2
IRS1
RESF1
LAMA4
MBOAT2
JAG1
LIF
FBXO32
RB1
LINC00958
RPLP0P2
ARLNC1
FILIP1
SH3RF1
EML2
TMEM132B
TIAM1
LINC01694
KCNAB2
FLNC
TSSC2
CELF2
TNFRSF25
SDF2L1
HINT2
RITA1
POLR3H
BDH1
LYPD1
TSEN54
GPN2
NOC4L
FASN
MIEN1
FXN
ISOC1
SNRNP25
CCDC137
RAB2A
TRAM1
ATP1B1
SCCPDH
SPRY4
ADGRE5
TNC
LIPG
LYPD5
ITGA10
PLXNA4
RHBDF2
IRAK2
PCK2
LTBP2
H6PD
SMAD3
DDR2
CD109
BACH1
MMP2
CD99L2
MMP9
ANXA3
PTS
ISCU
PPP5C
MTMR14
SDC1
CORO1B
TBCB
SIGMAR1
TNFSF15
PARL
GTF3A
DCUN1D5
BCL2A1
ADIPOR1
WDR18
PLXNA1
ARL4C
SLC44A1
MYLK
LOXL3
STEAP3
IL1B
PTPN14
COL6A3
UAP1
CEMIP
SERPINE1
TGM2
CD44
PAEP
FN1
HT1080 WT R4
HT1080 WT R2
HT1080 WT R3
HT1080 WT R1
HT1080 FUS-DDIT3 R3
HT1080 FUS-DDIT3 R1
HT1080 FUS-DDIT3 R2
HT1080 FUS-DDIT3 ruxolitinib R1
HT1080 FUS-DDIT3 ruxolitinib R4
HT1080 FUS-DDIT3 ruxolitinib R3
HT1080 FUS-DDIT3 ruxolitinib R2

Supplementary Figure 4. Heatmap with normalized expression of the 126 genes that were significantly regulated by FUS-DDIT3 expression and ruxolitinib treatment. Expression values are normalized counts from DESeq2 analysis of RNA sequencing data (1). R1-R4 represent biological replicates.

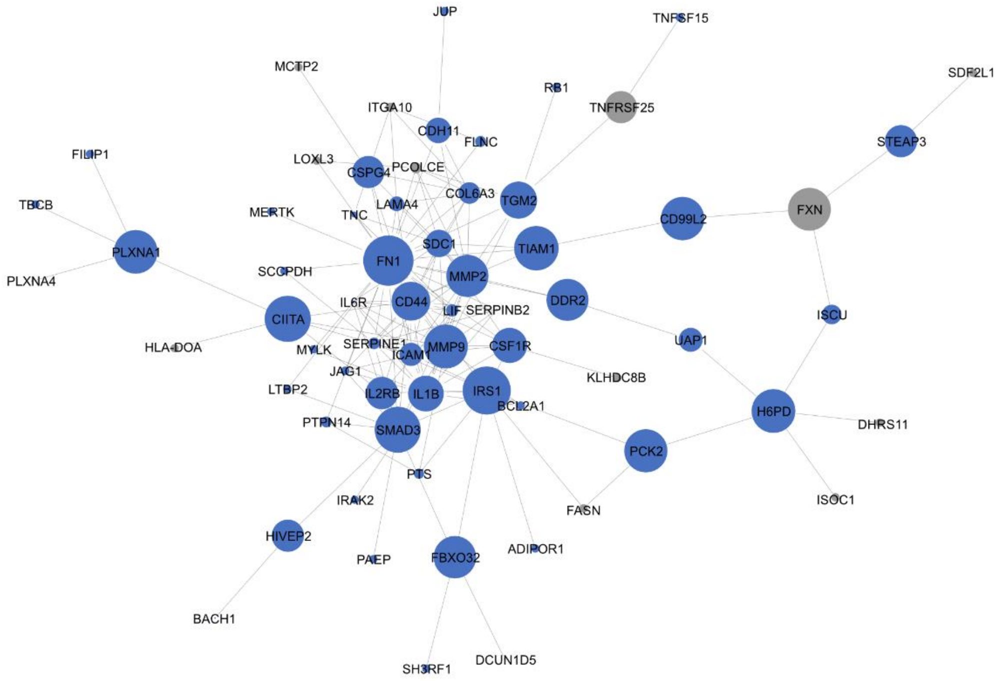

Supplementary Figure 5. Interaction network created with Cytoscape (2) based on protein interaction data retrieved from STRING (3). Node size is based on betweenness centrality. Nodes marked in blue indicate FUS-DDIT3-bound genes.

References

1. Love MI, Huber W, Anders S. Moderated estimation of fold change and dispersion for RNA-seq data with DESeq2. *Genome Biology* (2014) 15(12). doi: 10.1186/s13059-014-0550-8.

2. Shannon P, Markiel A, Ozier O, Baliga NS, Wang JT, Ramage D, et al. Cytoscape: a software environment for integrated models of biomolecular interaction networks. *Genome research* (2003) 13(11):2498-504. Epub 2003/11/05. doi: 10.1101/gr.1239303. PubMed PMID: 14597658; PubMed Central PMCID: PMCPMC403769.

3. Szklarczyk D, Gable AL, Lyon D, Junge A, Wyder S, Huerta-Cepas J, et al. STRING v11: protein-protein association networks with increased coverage, supporting functional discovery in genome-wide experimental datasets. *Nucleic Acids Res* (2019) 47(D1):D607-d13. Epub 2018/11/27. doi: 10.1093/nar/gky1131. PubMed PMID: 30476243; PubMed Central PMCID: PMCPMC6323986.
